# Identifying prognostic pairwise relationships among bacterial species in microbiome studies

**DOI:** 10.1371/journal.pcbi.1009501

**Published:** 2021-11-09

**Authors:** Sean M. Devlin, Axel Martin, Irina Ostrovnaya

**Affiliations:** Department of Epidemiology and Biostatistics, Memorial Sloan Kettering Cancer Center, New York, United States of America; DAL, CANADA

## Abstract

In recent literature, the human microbiome has been shown to have a major influence on human health. To investigate this impact, scientists study the composition and abundance of bacterial species, commonly using 16S rRNA gene sequencing, among patients with and without a disease or condition. Methods for such investigations to date have focused on the association between individual bacterium and an outcome, and higher-order pairwise relationships or interactions among bacteria are often avoided due to the substantial increase in dimension and the potential for spurious correlations. However, overlooking such relationships ignores the environment of the microbiome, where there is dynamic cooperation and competition among bacteria. We present a method for identifying and ranking pairs of bacteria that have a differential dichotomized relationship across outcomes. Our approach, implemented in an R package PairSeek, uses the stability selection framework with data-driven dichotomized forms of the pairwise relationships. We illustrate the properties of the proposed method using a published oral cancer data set and a simulation study.

## Author summary

Within an ecological system, microbial communities represent complex relationships between bacteria, where they co-exist and interact with each other in multiple ways including cooperation and competition. Most existing statistical tools for examining the association between microbiota and a disease state, such as individuals with and without cancer, focus on individual bacterium in isolation, ignoring the dynamic environment in which it lives. In this manuscript, we propose an algorithm for assessing the association between pairs of bacteria and a disease state. The approach provides a mechanism to rank pairs of bacteria, from pairs with the most evidence of an association with the disease state to the least amount of evidence. This ranking helps generate hypotheses and prioritize bacteria for further investigation. We illustrate the algorithm using a publicly available data set of oral cancer patients.

## Introduction

Recent technology has allowed for rapid and efficient evaluation of microbiome composition in human samples [1]. The presence and abundance of various bacteria has been shown to correlate with the onset of various diseases or disease characteristics. To date, the gut or oral microbiome has been associated with obesity and diabetes [2], cardiovascular disease [3], cancer [4], and many other conditions [1], as well as response to treatments [5]. While many statistical methods are available to evaluate differential abundance by a disease state, these methods commonly evaluate the association of a single bacterium at a time [6–10]. On the other hand, most methods that evaluate microbial networks and patterns of co-occurrence and co-exclusion are not designed to find how these patterns are associated with a disease or outcome [11–13]. In this paper, we propose a statistical framework for evaluating whether dichotomized interactions between bacterial species measured at a single time point are associated with a disease state.

Bacteria co-exist in complex ecological systems [11], where they interact in multiple ways including cooperation and competition. We will focus on the model where in presence of a disease state, the relationship between two bacterial species is modified. We seek to identify pairs of bacteria that have differing patterns of abundances in patients with and without disease, e.g., in the group of cases the abundance of one bacterium is higher while the abundance of the other is lower, compared to a non-diseased or control group. Our framework allows us to specify the dichotomized functional form of the relationship between the two bacteria.

The data for each sample are commonly obtained by amplicon sequencing, e.g. 16S rRNA gene sequencing, and consist of counts of reads attributed to bacterial species. The following three properties are common for such data that guide the choice of the statistical method: the data are high-dimensional yet sparse, due to many bacterial species being absent in a high proportion of samples; the data are compositional in nature, since sequencing coverage determines the total count; and the data are highly skewed. We propose a method to identify differential pairwise relationships among pairs of bacteria, modelled in a way that is robust given these data properties. We create a dichotomized variable for each pair that takes value 1 if one bacterium’s abundance is at least *c* fold higher than another bacterium’s abundance, where *c* is data-defined. The dichotomized comparison is robust in the presence of skewness and does not require prior normalization of the read counts since they compare abundances only within the same sample.

Other authors proposed alternative transformations of the microbiome counts including the Aitchison family of transformations [14], Isometric Logratio Transformation [15] and phylogenetic transform PhILR [16]. The transformations can be used within more traditional models designed to discover interactions. For example, random forests, although mainly used to build predictive models, can also be used to assess importance of the pairwise associations using one of these transformations [17]. The downside of this and other general methods for discovering interactions is that they are not designed specifically for microbiome data and might fail with the level of sparsity usually observed, and the functional form of the fitted interaction is not designed to find the specific relationship outlined in this paper.

Substantial correlation can be induced when creating pairwise relationships since any one bacterium is used to define (*p* − 1) pairs, where *p* is total number of bacterial species. To address this we propose to analyze all possible pairs of bacteria using a modification of stability selection [18]. Stability selection is a variable selection technique that utilizes multivariable penalized regression models like LASSO (least absolute shrinkage and selection operator) [19] fitted on resampled data. We propose to calculate the dominance score for each bacterial pair, defined as the percent of models fitted on resampled data where the pair had a non-zero coefficient. An additional advantage of the resampling approach is that the optimal threshold *c* can be identified in the out-of-sample data within each iteration. While there are no specialized algorithms for finding differential pairwise relationships to our knowledge, we will compare the proposed methodology to an analysis of single pairwise comparisons with False Discovery Rate (FDR) correction [20] along with the variable importance approach using random forests.

## Materials and methods

### Stability selection

We first introduce a general algorithm, which will be subsequently modified to estimate dominance scores. These scores are similar to the selection probabilities used in the stability selection algorithm [18]. We will outline how such scores can be used to evaluate dichotomized bacterial relationships. We propose to modify the stability selection algorithm by omitting the pre-specified tuning parameters for error control, as exchangeability is required for the error control framework. Instead, as outlined below, the penalty parameter for the degree of shrinkage in LASSO is selected randomly at each iteration.

Suppose the data consist of binary outcome *Y*_*k*_, for the *k*-th subject, *k* = {1, …, *n*}, and the *Z*_*ik*_ is the *i*-th predictor on *k*-th subject, *i* = {1, …, *p*}. The general algorithm is as follows:

1. On the *b*-th iteration, *b* = 1, …, *B*, take a random subsample *D* without replacement of subjects {1, …, *n*} of size ⌊*n*/2⌋. Using this subsample fit the LASSO model where (*β*_0_, *β*) are estimated by minimizing the LASSO functional:
  $$\begin{array}{c} -[\frac{1}{\left\lfloor n/2\right\rfloor }\sum_{k\subseteq D}Y_{k}\cdot \left(\beta_{0}+Z_{k}^{T}\beta \right)-\text{log}\left(1+e^{\left(\beta_{0}+Z_{k}^{T}\beta \right)}\right)]+{\lambda \|\beta \|}_{1} \end{array}$$
  using a regularization parameter λ selected at random from a standard LASSO penalty grid as previously described [21]. The grid usually spans 100 equally spaced points between the smallest value for which all estimated coefficients are zero and 0.001 times that value. Let $S_{i}^{b}$ be an indicator variable equal to 1 if the *i*-th variable has a non-zero coefficient in a fitted LASSO model in iteration *b*.
2. Repeat step 1 *B* times. Define the score $S_{i}=\frac{1}{B}\sum_{b=1}^{B}S_{i}^{b}$ as the proportion of B iterations where the *i*-th variable was selected. Variables are ranked by *S*_*i*_ which represent the strength of their association with the outcome; variables selected in at least *S*_*thr*_ of the models, i.e. *i*: *S*_*i*_ ≥ *S*_*thr*_, are considered to be the signals. *S*_*thr*_, the selection probability threshold, is usually in the range 70–90% and chosen *a priori*.

While we utilize the LASSO to define the dominance score in our algorithm, it can be formulated for other regularization procedures that induce sparsity.

### Proposed PairSeek method

The goal of the method is to find pairs of bacteria that have differential dichotomized relationship between cases and controls. For an introduction imagine the prevalence of two bacteria plotted against each other. Using the proposed PairSeek algorithm we aim to detect pairs of bacteria where a regression line through the origin with a slope *c* separates cases and controls. In other words, cases have relatively more, by a degree, of one bacterium than another, while controls have the opposite.

We will utilize resampling to concurrently identify the slope *c* and obtain a more stable measure of the association between each pair and the case status. At each resample, the patients are split into two groups randomly: one group to identify the optimal slopes *c* for each pair of bacteria, and then fit LASSO on the opposite set of patients with all possible pairs dichotomized at each respective *c* and random penalty parameter. After repeating these steps, we will calculate how often each pair of bacteria was selected among these LASSO runs. This quantity, which we call the dominance score, can be used to rank pair’s association with cohort. Below is the more detailed description of the proposed algorithm.

Let *X*_*ik*_ be the abundance of *i*-th bacterium, *i* = {1, …, *p*} in the *k*-th subject, *k* = {1, …, *n*}. For amplicon sequencing, *X*_*ik*_ would be the non-normalized number of reads matching the specific bacterial sequence. Suppose we want to examine whether the relationship between two bacterial abundances is dependent on some binary disease state *Y*_*k*_, e.g. cancer vs control.

We define binary indicator variables $Z_{k}^{ij}\left(c\right)=1\left(X_{ik}\leq cX_{jk}\right)$ which take a value of 1 if *X*_*jk*_ is at least *c*−fold smaller than *X*_*ik*_. We are looking for pairs that have *X*_*ik*_ ≤ *cX*_*jk*_ equal to 1, for example, in most cases and equal to 0 for most controls. If *c* is set to 1, the resulting dichotomized variables will only be useful for comparing bacteria with abundances on the same scale. Varying *c* allows more flexibility, so that bacteria with different prevalence can be compared.

Note that it is not necessarily the case that $Z_{k}^{ij}\left(c\right)=1-Z_{k}^{ji}\left(c\right)$, although often these might be equal. To avoid the redundancy only one pairwise comparison per *i*, *j* pair is fitted in each stability selection run. In order to assure that our method is invariant to the order of the variables we define the variables for each pair *i*, *j* based on order (*i*, *j*) or (*j*, *i*) randomly within each stability selection run.

The dominance score for the pair of bacteria *i*, *j* is calculated using the proposed PairSeek algorithm:

1. On the *b*-th iteration, starting from *b* = 1, take a random subsample *D* of size ⌊*n*/2⌋ without replacement from subjects {1, …, *n*}.
2. Randomly pick if $Z_{k}^{ij}\left(c\right)$ or $Z_{k}^{ji}\left(c\right)$ will represent pair *i*, *j* in the iteration *b*. Below without loss of generality we will use $Z_{k}^{ij}\left(c\right)$.
3. Using the out-of-sample complementary subset D* choose the optimal $c_{ij}^{b}$ as follows. First, denote the proportion that satisfy the inequality *X*_*ik*_ < *cX*_*jk*_ who are cases as $p_{1}^{ij}\left(c\right)=\frac{\sum_{k\subseteq D^{*}}Y_{k}Z_{k}^{ij}\left(c\right)}{\sum_{k\subseteq D^{*}}Z_{k}^{ij}\left(c\right)}$, and denote the proportion that do not satisfy the inequality who are cases as $p_{2}^{ij}\left(c\right)=\frac{\sum_{k\subseteq D^{*}}Y_{k}\left(1-Z_{k}^{ij}\left(c\right)\right)}{\sum_{k\subseteq D^{*}}\left(1-Z_{k}^{ij}\left(c\right)\right)}$.
  Let $\gamma^{ij}\left(c\right)=\frac{1}{|D^{*}|}\sum_{k\subseteq D^{*}}Z_{k}^{ij}\left(c\right)$. The optimal $c_{ij}^{b}$ is determined by minimizing the entropy:
  $$\begin{array}{c} {}[\gamma^{ij}\left(c\right)p_{1}^{ij}\left(c\right)\left(1-p_{1}^{ij}\left(c\right)\right)+\left(1-\gamma^{ij}\left(c\right)\right)p_{2}^{ij}\left(c\right)\left(1-p_{2}^{ij}\left(c\right)\right)]. \end{array}$$
  To find the optimal value of $c_{ij}^{b}$ we calculate the entropy above on a grid of potential values between 20-th and 80-th percentiles of *X*_*ik*_/*X*_*jk*_.
  If *X*_*ik*_ = *X*_*jk*_ = 0, $Z_{k}^{ij}\left(c\right)$ is drawn randomly from a binomial distribution with probability 0.5.
4. Fit the LASSO model with outcome *Y*_*k*_ and all pairwise variables $Z_{k}^{ij}\left(c_{ij}^{b}\right)$ in the subset of the samples *D* with randomly drawn regularization parameter λ. Let $S_{ij}^{b}$, *i* < *j* be the indicator function equal to 1 when *i*, *j*-th pair is selected in sampling step *b*.
5. Repeat steps 1–4 *B* times. The dominance score, defined as $S_{ij}=\frac{1}{B}\sum_{b=1}^{B}S_{ij}^{b}$, for each pair *i* < *j* is the proportion of iterations where the pair has a non-zero coefficient across the *B* iterations.

We propose to rank pairs based on their dominance scores and select the ones with scores above the prespecified threshold *S*_*thr*_. This algorithm is a tool for ranking pairwise relationships; selecting pairs satisfying *S*_*thr*_ is used for defining the set of “promising” pairs for further investigation. Higher values of *S*_*thr*_ would result in fewer “signals”, but also fewer false positives.

## Results

### Application to an oral cancer data set

We applied our method to 16S rRNA gene sequencing data on 121 oral cancer patients and 242 normal controls previously analyzed [22]. After aggregating bacterial counts from 2770 operational taxonomic units (OTUs) to the genus taxonomic level and filtering out genera observed in fewer than 10% of the samples, a total of 61 genera were included in the analysis. Note that this filtering step is completely independent of the outcome.

**Fig 1:**
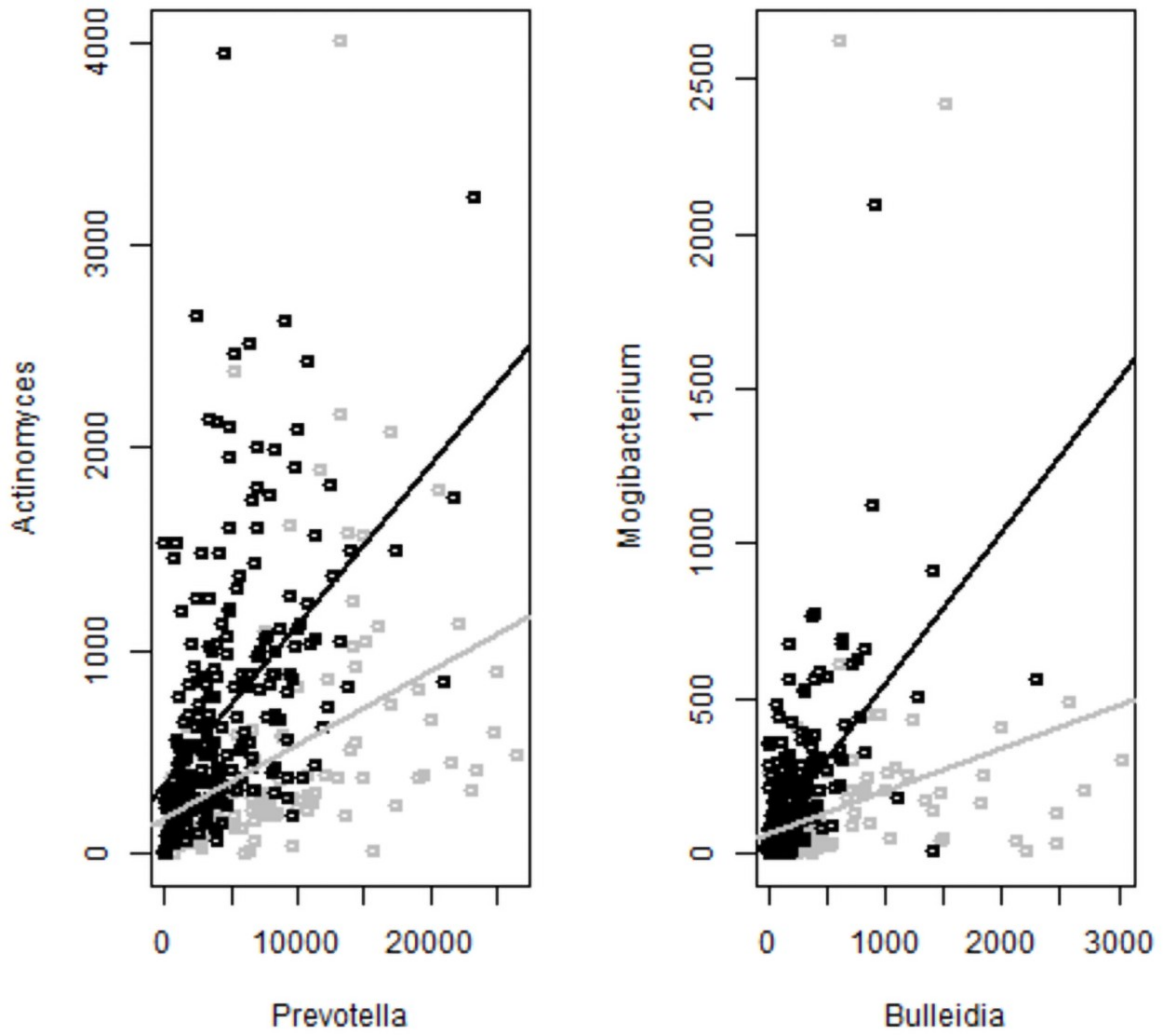
Two pairs of bacteria with a differential relationship between oral cancer cases (red) and healthy controls (black). Black and red lines represent linear regression lines fitted within cases and controls separately.

When we applied PairSeek to this dataset, there were only two pairs with dominance scores above 80%: Prevotella/Actinomyces (88%), and Bulleidia/Mogibacterium (82%). Fig 1 illustrates these relationships. The next highest dominance score was 72%.

The bacteria in these two pairs were identified in previous colon cancer studies. In particular, Prevotella, Bulleidia, Mogibacterium were found to be associated with colon cancer [23, 24] and Prevotella co-aggregates with Actinomyces as previously described [25].

For comparison we also performed an analysis of these data using simple logistic regression. We analyzed individual pairs of bacteria (not transformed to relative abundance) dichotomized using a fitted linear regression line between the two bacteria, regardless of whether the slope of this regression line was significantly different from zero. To align with PairSeek, no intercept was fit in each linear regression model so that each fitted line went through the origin. The association with the outcome was evaluated using a separate logistic regression for each pair. This minimized over-fitting in the absence of external data to estimate an outcome-based dichotomization threshold, yet still avoids data normalization. There were 605/1830 pairs with p-values ≤ 0.05, and 370 remained significant after FDR adjustment at 0.05. If the model was adjusted for the relative abundance of the two corresponding main effects, then a total of 366 pairs among 1830 had a p-value ≤ 0.05, and 76 remained significant after FDR adjustment. If the two corresponding main effects were alternatively modeled using a centered log-ratio transformation (CLR), then 273 and 33 pairs were significant without and with FDR adjustment, respectively.

One explanation for this high number of significant dichotomized pairs is the induced correlation between pairwise markers. This can inflate the rate of significant pairs and also invalidate the FDR adjustment [26]. Indeed, the absolute value of the log odds ratio between pairwise markers dichotomized at regression line that share the same genus as one component of the pair, e.g. 1,2 and 1,3, were higher than those from the non-overlapping pairs, e.g. 1,2 and 3,4 (medians of absolute values of the log odds ratios were 0.86 and 0.55, respectively).

### Simulations

While the previous section identified two pairs of bacteria with a differential relationship across cancers and controls, the accuracy of these results is uncertain due to the absence of a gold-standard or known true relationship. To further evaluate the performance of the proposed algorithm, we designed a data-driven simulation experiment. The actual oral cancer genus-level count data described in previous section with 61 genera and 1,830 corresponding pairs were used to evaluate the operating characteristics, while the outcome (cancer/control status) was simulated using these data as described below.

#### Outcome generation

The binary outcome was generated under three general scenarios: Alternative, Null 1, and Null 2. Under the Alternative, either *M* = 2 or *M* = 6 pairs of bacteria were associated with the outcome. Under Null 1, either *M*_0_ = 2 or *M*_0_ = 6 individual bacteria were associated with the outcome as main effects, without any interactions between bacteria. Lastly, under Null 2, all bacteria were independent of the outcome.

Under the **Alternative scenario** with *M* = 2, the two pairs identified in the previous section were used for outcome generation. Let the abundance of Bulleidia be denoted as $X_{1}^{1}$, Mogibacterium as $X_{1}^{2}$, and Prevotella as $X_{2}^{1}$, and Actinomyces as $X_{2}^{2}$. With *M* = 6, four additional pairs were selected, which were unique bacteria whose pairing had relatively low correlation with the first two pairs.

Using either of the two *M* values, the binary outcome *Y* was generated according to the standard logistic model:

$$\begin{array}{ccc} Y & ∼ & \text{Binomial}(\pi )\\ \text{logit}(\pi ) & = & \beta_{0}+\sum_{m=1}^{M}\beta_{m}1\left\{X_{m}^{1}\leq c_{m}X_{m}^{2}\right\} \end{array}$$ (1)

where logit(*π*) = log(*π*/(1 − *π*)). The value *c*_*m*_ was selected by minimizing the entropy for the pairing using the actual case-control outcome in the data analysis.

For scenarios with *M* = 2, values of *β*_1_ = 2.56 and *β*_2_ = 2.28 were used, as estimated using the true case-control outcome. The value of *β*_0_ was selected such that the average logit(*π*) was centered at 0.

For the scenario with *M* = 6, the setup was simplified such that *β*_*m*_ = −2.25 or 2.25, with the direction of the effect selected based on the observed direction for the true case-control outcome.

Under **Null 1 scenario**, the top two or six individual genus-level bacteria with the largest coefficients (main effects) were selected using a single regularized regression with a LASSO penalty and the true case-control outcome from the data analysis. For the purpose of choosing these main effects the data were converted to the relative abundance scale by dividing by the per-patient total read count and further filtered to the subset of bacteria observed in at least 40% of the patients. For *M*_0_ = 2 or 6, the data were generated as $\text{logit}\left(\pi \right)=\beta_{0}+\sum_{m=1}^{M_{0}}\beta_{m}X_{m}^{R}$, where $X_{m}^{R}$ corresponds to *X*_*m*_ standardized to the relative abundance scale. For all simulations in Null 1, *β*_*m*_ = −0.7, which is the largest coefficient from the LASSO model; all six individual coefficients from the LASSO model had a negative estimated log-odds ratio. Similar to before, *β*_0_ was selected such that the average logit(*π*) was centered at 0.

Finally, for **Null 2 scenario**, no main effect or pairwise marker affected the outcome, and the outcome was generated as *Y*∼ Binomial(*π* = 0.5).

#### Metrics for methods comparison

We compared the proposed method to the performance of logistic regression with a single pairwise marker that was dichotomized at the estimated linear regression line. Each respective regression model was adjusted for the two main effects using the relative abundance (RA) or centered log-ratio transformation (CLR) scales.

For each individual *k*, the binary variable was created by $X_{ik}>{\hat{d}}_{ij}X_{jk}$, where ${\hat{d}}_{ij}$ was the estimated coefficient taken directly from the $\left(\begin{array}{c} p\\ 2 \end{array}\right)$ linear regression models. We did not filter pairs based on the Wald test associated with each coefficient. This initial step is independent of the outcome *Y*. We envision scenarios when the Wald test for ${\hat{d}}_{ij}$ is not significant, but the pair $X_{ik}>{\hat{d}}_{ij}X_{jk}$ is associated with *Y* and vice versa. The reason why pairs were dichotomized at the linear regression line is that, unlike within the proposed method, there is no way to choose the threshold on out-of-sample data, and the regression line is an unbiased way to estimate a threshold independent of the outcome.

The operating characteristics were evaluated by estimating the average number of true and false positives. For our algorithm, the thresholds for the dominance score of each pair were 0.7, 0.8, or 0.9, and for the single pair analysis, significance was defined as FDR levels below 0.05, 0.01, 0.001, or 0.0001. Since the two methods are not calibrated based on the number or rate of true or false positives, we also compared the relative ranking of the true pairs of bacteria associated with outcome.

Lastly, we compared the performance of the proposed method to pairwise variable importance using random forests [17]. The data used in the forest were on the relative abundance scale. There is no corresponding threshold to declare true and false positive pairs using this approach. However, this method was included when comparing the relative ranking of the true pairs associated with outcome.

#### Simulations results

**Fig 2:**
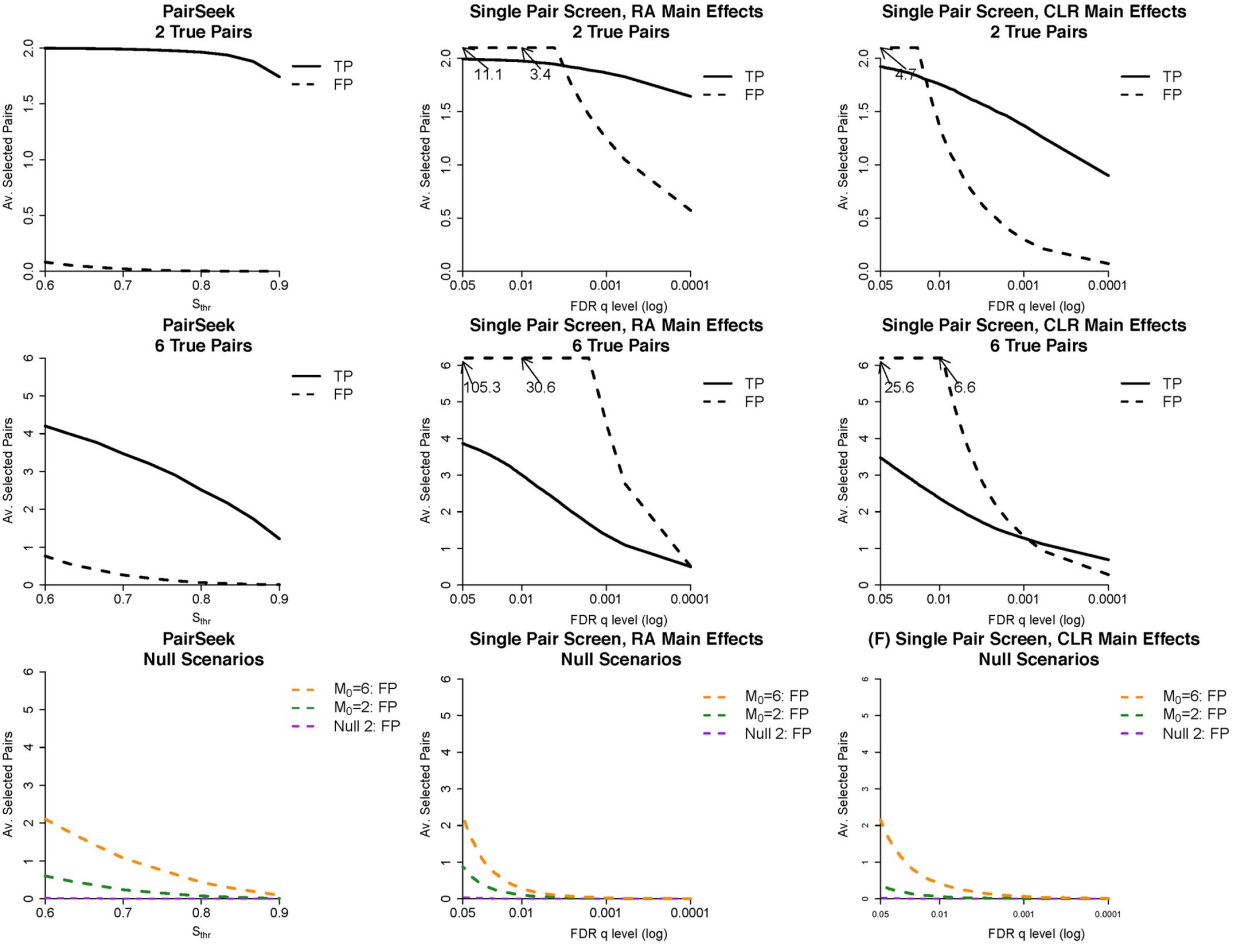
Operating characteristics of PairSeek and the FDR-adjusted single pair screens, which adjusted for main effects using either relative abundance (RA) or the centered log-ratio transformation (CLR). The top row shows the average number of true and false positives (TP and FP, respectively) across different values of the selection threshold for *M* = 2 under the Alternative scenario for PairSeek along with the two single pair screens for different FDR thresholds. The second row are parallel results for M = 6. The last row shows the performance of the three methods for scenarios Null 1 (*M*_0_ = 2 or 6 main effects only) and Null 2 (no association between the microbiome and generated outcome).

Fig 2 shows the operating characteristics of PairSeek and the two FDR adjusted [20] screens of single bacterial pairs, which adjusted for the main effects using relative abundance or CLR. Table A in S1 Supporting Information, Table B in S1 Supporting Information, Table C in S1 Supporting Information, and Table D in S1 Supporting Information illustrate the same information in numeric format. The top row of Fig 2 displays the average true and false positives for PairSeek for different *S*_*thr*_ thresholds in the Alternative scenario. With two true pairs (*M* = 2), *S*_*thr*_ thresholds of 0.7, 0.8 and 0.9 on average identified 1.99, 1.96, and 1.73, respectively, of the two hits, while having on average ≤ 0.02 false pairs with dominance score above any of these thresholds. The relative performance with six true pairs (*M* = 6) was somewhat poorer with on average 1.22 to 3.47 true positives out of 6 for *S*_*thr*_ thresholds of 0.7 to 0.9, with ≤ 0.26 on average false positives. These results are contrasted with the two single pair screens using FDR-correction. These methods were able to identify a similar number of true positives for M = 2 or 6; however, the number of false positives associated with the methods were higher, at a magnitude high enough to question the utility of the approach. As a note to this latter analysis, if a standard multiplicative interaction was alternatively fit using logistic regression, this would result in a substantial drop in performance; this is expected as this approach aims to identify a different type of association than the one used in the simulation construction (Table E in S1 Supporting Information).

Results of the Null 1 and Null 2 scenarios are presented in last row of Fig 2 for the three analyses. Under Null 1, PairSeek had a minimal number of false positives selected on average; similar to the Alternative scenario, the largest false positive average was 1.08 for *S*_*thr*_ = 0.7. The approach of the single pair screens had a similar low number of false positives, particularly for more stringent cut-offs. Both PairSeek and the single pair analyses performed well in Null scenario 2.

The average rankings of the true pairs used to generate the binary outcome among all potential pairs are provided in Box 1 for the Alternative scenario. When two pairs were used to generate the outcome, the top two pairs, on average, in PairSeek were the true pairs. The top pair was a true pair, on average, for the two FDR screens and the second true pair ranked 2.5–3.1. When the outcome was generated based on six underlying pairs, PairSeek performed well with a median rank among the six underlying pairs of approximately 3.8. This is contrasted to the two FDR screens, which median rank ranged from 89.5–283.6.

**Box 1. Average ranking of the underlying true pairs used to generate the outcome in the Alternative scenario simulation.**

**Table:** 

PairSeek			Single Pair Screen, RA Main Effects		
1^st^ True Hit	2^nd^ True Hit	Median Rank	1^st^ True Hit	2^nd^ True Hit	Median Rank
2 Pairs			2 Pairs		
1.0	2.0		1.0	2.5	
6 Pairs			6 Pairs		
1.0	2.0	3.8	2.0	9.2	283.6
Single Pair Screen, CLR Main Effects			Random Forests		
1^st^ True Hit	2^nd^ True Hit	Median Rank	1^st^ True Hit	2^nd^ True Hit	Median Rank
2 Pairs			2 Pairs		
1.1	3.1		11.5	222.9	
6 Pairs			6 Pairs		
1.3	5.1	89.5	24.7	71.6	250.1

Random forests were also used to rank the relative importance of each pair. However, the two or six true pairs were ranked lower using this approach than either PairSeek or the single pair screens.

## Discussion

In summary, we propose a novel algorithm to identify pairwise relationships between bacteria that are associated with an outcome. There are several advantages to this method. We bypass the need for data normalization since the comparisons are made within sample. This avoids having to adjust for a compositional data structure as would be the case in other regression settings [9]. Additionally, the sample splitting within each iteration of the algorithm allows for the estimation of the dichotomization threshold outside of the subsample analyzed; alternatively, *c* can be set to 1 if only bacteria on the same scale are of interest. The outlined method could also be easily extended to estimate other parametric functions of the two bacterial abundances in place of $Z_{k}^{ij}\left(c\right)$.

We compared the proposed method to an approach of having separate regression models for each dichotomized pair of bacteria, and our method demonstrates substantially lower false positives. This may be due in part to induced correlations between pairwise markers. Such pairs are more likely to be eliminated in our proposed framework through the selected penalized model.

The proposed method estimates the “optimal” threshold on out-of-sample data; it is not possible to do this in the single pair approach, so alternatively, the pairwise relationships are dichotomized using a fitted linear regression line. If an outcome-based threshold were selected in this approach, the average true positives would likely increase, but at a cost of higher false positives due to over-fitting.

In order to identify pairs as promising, a dominance score threshold must be selected in an informal or arbitrary manner. Simulations demonstrated that a threshold between 70%-80% leads to reasonable rates of true and false positives. This estimated dominance score is a way to rank and prioritize pairs of bacteria, and the threshold provides a rule to identify pairs with a corresponding strong signal. However, this approach does not provide a formal error control argument, such as in stability selection.

Although LASSO is used in the manuscript, other penalized regression models may be substituted. One feature of LASSO is that, if *p* > >*n*, at most *n* variables are selected in each run. Even though in the stability selection framework more than *n* variables can possibly reach high selection probability, this selection of *n* maximum variables on a single run may be a limitation. The proposed framework is most useful when relatively few significant pairwise associations are expected.

This algorithm is developed for microbiome data collected at the single time point; it is based on a cross-sectional view of the differential relationship between pairs of bacteria for cases and controls. To ascertain the degree of competitiveness between two bacteria, longitudinal samples leading up to the time of the incident case development may be required. Unfortunately, in many clinical scenarios, such data do not exist. The cross-sectional snapshot utilized in our method, however, provides a mechanism to prioritize pairs of bacteria for further investigation.

Another paper published by McGregor et al [27] tackles a similar problem of estimating differential co-occurrence networks in microbiome studies. In their method, a covariance matrix is specified using a Bayesian model to be a function of a covariate, such as case/control status. While their goal is similar, their algorithm is designed to find a different type of relationship, such as pairs that have different strengths of correlation in cases and controls. This objective is different from PairSeek′s: a pair of bacteria may be uncorrelated for cases and controls, but there may exists a *c* that perfectly separates cases and controls in the covariate space. Alternatively, cases and control may have differential correlation, but no *c* would exist to separate case status. In addition, their method utilizes a more complex mathematical model and requires specification of priors. Both methods could be used jointly in a data analysis and might provide different and complementary insights into disease related bacterial relationships.

While the algorithm as presented is for a binary outcome, it is straightforward to extend our method to continuous or time-to-event outcomes using penalized linear or Cox proportional hazards regression, both of which are implemented in R. We anticipate that PairSeek will help unravel important relationships among bacteria that are associated with these additional outcomes.

## Supporting information

S1 Supporting Information **Table A. Alternative scenario—PairSeek** Average true positives (T.P.) and false positives (F.P.) at various thresholds *S*_*thr*_. **Table B. Alternative scenario—single pair approach**. Average true positives (T.P.) and false positives (F.P.) for the screen of individual dichotomized pairs at different FDR levels. Main effects were included in each model using either relative abundance (RA) or centered log-ratio transformation (CLR). **Table C. Null scenarios—PairSeek**. Average false positives (F.P.) at various thresholds *S*_*thr*_ in the Null 1 and Null 2 simulation scenario. **Table D. Null scenarios—single pair approach**. Average false positives (F.P.) for the screen of individual dichotomized pairs at different FDR levels in the Null 1 and Null 2 scenarios. Main effects were included in each model using either relative abundance (RA) or centered log-ratio transformation (CLR). **Table E. Alternative scenario—single pair approach using multiplicative interactions**. Average true positives (T.P.) and false positives (F.P.) when a standard multiplicative interaction was fit to each pair using logistic regression. The main effects and interaction terms were included in each model using either relative abundance (RA) or centered log-ratio transformation (CLR).
(PDF)

## References

[1] Cho I, Blaser MJ. The human microbiome: at the interface of health and disease. Nature Reviews Genetics. 2012;13(4):260–270. doi: 10.1038/nrg3182 22411464 PMC3418802

[2] Hartstra AV, Bouter KEC, Bäckhed F, Nieuwdorp M. Insights Into the Role of the Microbiome in Obesity and Type 2 Diabetes. Diabetes Care. 2015;38(1):159–165. doi: 10.2337/dc14-0769 25538312

[3] Howitt MR, Garrett WS. A complex microworld in the gut: Gut microbiota and cardiovascular disease connectivity. Nature Medicine. 2012. doi: 10.1038/nm.2895 22869188

[4] Goodman B, Gardner H. The microbiome and cancer. The Journal of Pathology. 2018;244(5):667–676. doi: 10.1002/path.5047 29377130

[5] Routy B, Le Chatelier E, Derosa L, Duong CPM, Alou MT, Daillère R, et al. Gut microbiome influences efficacy of PD-1-based immunotherapy against epithelial tumors. Science (New York, NY). 2018;359(6371):91–97. doi: 10.1126/science.aan3706 29097494

[6] Li H. Microbiome, Metagenomics, and High-Dimensional Compositional Data Analysis. Annual Review of Statistics and Its Application. 2015;2(1):73–94. doi: 10.1146/annurev-statistics-010814-020351

[7] phyloseq: An R Package for Reproducible Interactive Analysis and Graphics of Microbiome Census Data;. Available from: http://journals.plos.org/plosone/article?id=10.1371/journal.pone.0061217. 10.1371/journal.pone.0061217 PMC3632530 23630581

[8] Zhan X, Plantinga A, Zhao N, Wu MC. A fast small-sample kernel independence test for microbiome community-level association analysis. Biometrics. 2017;73(4):1453–1463. doi: 10.1111/biom.12684 28295177 PMC5592124

[9] Lu J, Shi P, Li H. Generalized linear models with linear constraints for microbiome compositional data. Biometrics. 2019;75(1):235–244. doi: 10.1111/biom.12956 30039859

[10] Lin W, Shi P, Feng R, Li H. Variable selection in regression with compositional covariates. Biometrika. 2014;101(4):785–797.

[11] Faust K, Sathirapongsasuti JF, Izard J, Segata N, Gevers D, Raes J, et al. Microbial Co-occurrence Relationships in the Human Microbiome. PLOS Computational Biology. 2012;8(7):e1002606. doi: 10.1371/journal.pcbi.1002606 22807668 PMC3395616

[12] Tsai KN, Lin SH, Liu WC, Wang D. Inferring microbial interaction network from microbiome data using RMN algorithm. BMC Systems Biology. 2015;9:54. doi: 10.1186/s12918-015-0199-2 26337930 PMC4560064

[13] Lo C, Marculescu R. MPLasso: Inferring microbial association networks using prior microbial knowledge. PLOS Computational Biology. 2017;13(12):e1005915. doi: 10.1371/journal.pcbi.1005915 29281638 PMC5760079

[14] Aitchison J. The Statistical Analysis of Compositional Data. Journal of the Royal Statistical Society Series B (Methodological). 1982;44(2):139–177. doi: 10.1111/j.2517-6161.1982.tb01195.x

[15] Egozcue JJ, Pawlowsky-Glahn V, Mateu-Figueras G, Barceló-Vidal C. Isometric Logratio Transformations for Compositional Data Analysis. Mathematical Geology. 2003;35(3):279–300. doi: 10.1023/A:1023818214614

[16] Silverman JD, Washburne AD, Mukherjee S, David LA. A phylogenetic transform enhances analysis of compositional microbiota data. eLife;6. doi: 10.7554/eLife.21887 28198697 PMC5328592

[17] Ishwaran H. Variable importance in binary regression trees and forests. Electronic Journal of Statistics. 2007;1:519–537. doi: 10.1214/07-EJS039

[18] Meinshausen N, Bühlmann P. Stability selection. Journal of the Royal Statistical Society Series B (Statistical Methodology). 2010;72(4):417–473. doi: 10.1111/j.1467-9868.2010.00740.x

[19] Tibshirani R. Regression Shrinkage and Selection via the Lasso. Journal of the Royal Statistical Society Series B (Methodological). 1996;58(1):267–288. doi: 10.1111/j.2517-6161.1996.tb02080.x

[20] Benjamini Y, Hochberg Y. Controlling the False Discovery Rate: A Practical and Powerful Approach to Multiple Testing. Journal of the Royal Statistical Society Series B (Methodological). 1995;57(1):289–300. doi: 10.1111/j.2517-6161.1995.tb02031.x

[21] Friedman J, Hastie T, Tibshirani R. Regularization Paths for Generalized Linear Models via Coordinate Descent. Journal of Statistical Software. 2010;33(1):1–22. doi: 10.18637/jss.v033.i01 20808728 PMC2929880

[22] Börnigen D, Ren B, Pickard R, Li J, Ozer E, Hartmann EM, et al. Alterations in oral bacterial communities are associated with risk factors for oral and oropharyngeal cancer. Scientific Reports. 2017;7. doi: 10.1038/s41598-017-17795-z 29247187 PMC5732161

[23] Gao Z, Guo B, Gao R, Zhu Q, Qin H. Microbiota disbiosis is associated with colorectal cancer. Frontiers in Microbiology. 2015;6. doi: 10.3389/fmicb.2015.00020 25699023 PMC4313696

[24] Xu K, Jiang B. Analysis of Mucosa-Associated Microbiota in Colorectal Cancer. Medical Science Monitor: International Medical Journal of Experimental and Clinical Research. 2017;23:4422–4430. doi: 10.12659/MSM.904220 28904330 PMC5609654

[25] Nesbitt WE, Fukushima H, Leung KP, Clark WB. Coaggregation of Prevotella intermedia with oral Actinomyces species. Infection and Immunity. 1993;61(5):2011–2014. doi: 10.1128/iai.61.5.2011-2014.1993 8478088 PMC280796

[26] Schwartzman A, Lin X. The effect of correlation in false discovery rate estimation. Biometrika. 2011;98(1):199–214. 23049127 10.1093/biomet/asq075 PMC3412603

[27] McGregor K, Labbe A, Greenwood CMT. MDiNE: a model to estimate differential co-occurrence networks in microbiome studies. Bioinformatics (Oxford, England). 2020;36(6):1840–1847. 10.1093/bioinformatics/btz824 PMC7075537 31697315

