# Supplementary material for: Identifying prognostic pairwise relationships among bacterial species in microbiome studies
Source: PLoS Comput Biol. 2021 Nov 9;17(11):e1009501. doi: 10.1371/journal.pcbi.1009501 (PMC8631663; doi:10.1371/journal.pcbi.1009501)
Supplement: S1 Supporting Information — Table A. Alternative scenario—PairSeek Average true positives (T.P.) and false positives (F.P.) at various thresholds Sthr. Table B. Alternative scenario—single pair approach. Average true positives (T.P.) and false positives (F.P.) for the screen of individual dichotomized pairs at different FDR levels. Main effects were included in each model using either relative abundance (RA) or centered log-ratio transformation (CLR). Table C. Null scenarios—PairSeek. Average false positives (F.P.) at various thresholds Sthr in the Null 1 and Null 2 simulation scenario. Table D. Null scenarios—single pair approach. Average false positives (F.P.) for the screen of individual dichotomized pairs at different FDR levels in the Null 1 and Null 2 scenarios. Main effects were included in each model using either relative abundance (RA) or centered log-ratio transformation (CLR). Table E. Alternative scenario—single pair approach using multiplicative interactions. Average true positives (T.P.) and false positives (F.P.) when a standard multiplicative interaction was fit to each pair using logistic regression. The main effects and interaction terms were included in each model using either relative abundance (RA) or centered log-ratio transformation (CLR). (PDF) [file pcbi.1009501.s001.pdf]

S1 Supporting Information

Table A. Alternative scenario - PairSeek Average true positives (T.P.) and false positives (F.P.) at various thresholds S_{thr}.

Table B. Alternative scenario - single pair approach. Average true positives (T.P.) and false positives (F.P.) for the screen of individual dichotomized pairs at different FDR levels. Main effects were included in each model using either relative abundance (RA) or centered log-ratio transformation (CLR).

Table C. Null scenarios - PairSeek. Average false positives (F.P.) at various thresholds S_{thr} in the Null 1 and Null 2 simulation scenario.

Table D. Null scenarios - single pair approach. Average false positives (F.P.) for the screen of individual dichotomized pairs at different FDR levels in the Null 1 and Null 2 scenarios. Main effects were included in each model using either relative abundance (RA) or centered log-ratio transformation (CLR).

Table E. Alternative scenario - single pair approach using multiplicative interactions. Average true positives (T.P.) and false positives (F.P.) when a standard multiplicative interaction was fit to each pair using logistic regression. The main effects and interaction terms were included in each model using either relative abundance (RA) or centered log-ratio transformation (CLR).

Table A. Alternative scenario - PairSeek. Average true positives (T.P.) and false positives (F.P.) at various thresholds S_{thr}.

	2 True Pairs		6 True Pairs	
S_{thr}	T.P.	F.P.	T.P.	F.P.
0.7	1.99	0.02	3.47	0.26
0.8	1.96	0.00	2.51	0.06
0.9	1.73	0.00	1.22	0.01

Table B. Alternative scenario - single pair approach. Average true positives (T.P.) and false positives (F.P.) for the screen of individual dichotomized pairs at different FDR levels. Main effects were included in each model using either relative abundance (RA) or centered log-ratio transformation (CLR).

	RA Main Effects				CLR Main Effects			
FDR	2 True Pairs		6 True Pairs		2 True Pairs		6 True Pairs	
Level	T.P.	F.P.	T.P.	F.P.	T.P.	F.P.	T.P.	F.P.
0.05	1.99	11.14	3.87	105.32	1.92	4.75	3.48	25.61
0.01	1.98	3.36	3.01	30.57	1.76	1.36	2.37	6.62
0.001	1.86	1.25	1.36	4.41	1.37	0.30	1.28	1.33
0.0001	1.64	0.57	0.50	0.51	0.90	0.07	0.69	0.28

Table C. Null scenarios - PairSeek. Average false positives (F.P.) at various thresholds S_{thr} in the Null 1 and Null 2 simulation scenario.

S_{thr}	F.P.
Null 1: 2 Main Effects	
0.7	0.24
0.8	0.07
0.9	0.07
Null 1: 6 Main Effects	
0.7	1.08
0.8	0.44
0.9	0.44
Null 2: Complete Null	
0.7	0.00
0.8	0.00
0.9	0.00

Table D. Null scenarios - single pair approach. Average false positives (F.P.) for the screen of individual dichotomized pairs at different FDR levels in the Null 1 and Null 2 scenarios. Main effects were included in each model using either relative abundance (RA) or centered log-ratio transformation (CLR).

	RA Main Effects	CLR Main Effects
S_{thr}	F.P.	F.P.
Null 1: 2 Main Effects		
0.05	0.87	0.36
0.01	0.11	0.07
0.001	0.01	0.01
0.0001	0.00	0.00
Null 1: 6 Main Effects		
0.05	2.30	2.16
0.01	0.28	0.41
0.001	0.03	0.06
0.0001	0.00	0.01
Null 2: Complete Null		
0.05	0.04	0.02
0.01	0.00	0.00
0.001	0.00	0.00
0.0001	0.00	0.00

Table E. Alternative scenario - single pair approach. Average true positives (T.P.) and false positives (F.P.) when a standard additive interaction was fit to each pair using logistic regression. The main effects and interaction terms were included in each model using either relative abundance (RA) or centered log-ratio transformation (CLR).

	RA Main Effects and Interaction				CLR Main Effects and Interaction			
FDR	2 True Pairs		6 True Pairs		2 True Pairs		6 True Pairs	
Level	T.P.	F.P.	T.P.	F.P.	T.P.	F.P.	T.P.	F.P.
0.05	0.00	0.06	0.00	0.23	0.00	5.26	0.00	0.23
0.01	0.00	0.05	0.00	0.13	0.00	0.67	0.00	0.13
0.001	0.00	0.05	0.00	0.11	0.00	0.03	0.00	0.11
0.0001	0.00	0.05	0.00	0.11	0.00	0.03	0.00	0.11
